# Perioperative Care and Airway Management for a Patient With Sagliker Syndrome

**DOI:** 10.7759/cureus.10691

**Published:** 2020-09-28

**Authors:** QiLiang Chen, Javier Lorenzo, Amy Lu

**Affiliations:** 1 Department of Anesthesiology, Stanford University, Stanford, USA

**Keywords:** sagliker syndrome, perioperative anesthesia service, difficult airway management, perioperative care, case report, ecmo

## Abstract

In this report, we present a case of a patient with a history of complex airway anatomy secondary to Sagliker syndrome (SS) who presented with acute exacerbation of chronic respiratory failure. The patient’s difficult airway, complicated medical comorbidities, and poor psychosocial status posed a unique challenge for providing safe care during an emergency. The perioperative anesthesia service (PAS), led by critical care anesthesiologists, coordinated a multidisciplinary airway management plan. The PAS team also assisted this medically complex patient with her decision-making process.

A 37-year-old female with SS, which is characterized by irreversible disfiguring of head and neck anatomy secondary to end-stage renal disease (ESRD) and poorly controlled hyperparathyroidism, presented with acute exacerbation of chronic respiratory failure due to hypervolemia. The patient’s respiratory status rapidly deteriorated despite aggressive hemodialysis, requiring transfer to the ICU. Given the challenging anatomy and poor respiratory reserve in this patient, the PAS team helped coordinate a comprehensive airway plan that involved transnasal fiberoptic intubation, and in case of emergency, extracorporeal membrane oxygenation (ECMO) as a bridge to a surgical airway. During the decision-making process, the patient was found to be in psychological distress and had limited insights into her condition. The PAS team helped facilitated multidisciplinary goals-of-care discussions for the patient and her family. Fortunately, the patient’s oxygenation improved with noninvasive oxygen support and aggressive hemodialysis without the need for intubation. She was discharged with outpatient follow-up appointments arranged to discuss long-term management.

This is the first reported case of SS in the United States. The early involvement by the PAS team helped coordinate a multidisciplinary care plan for this patient with a difficult airway and complex comorbidities. This report highlights an innovative airway algorithm for a potentially “cannot-intubate, cannot ventilate" complex airway, and the PAS team’s role in providing support for the patient’s physical and psychological needs, suggesting that a comprehensive perioperative service can improve the quality and safety of care, not only for surgical patients but also for medically complex patients as well.

## Introduction

Sagliker syndrome (SS) is a rare condition related to end-stage renal disease (ESRD); it is characterized by a progressively disfiguring facial deformity, dental abnormalities, peripheral neuropathy, and high prevalence of psychiatric comorbidities [1-3]. The disease is thought to be primarily driven by hyperparathyroidism in the setting of ESRD [1,4]. Genes associated with plasma calcium-sensing and bone dysplasia have also been implicated in disease pathogenesis [5,6]. These bony changes will irreversibly worsen if the parathyroid hormone (PTH) level is inadequately controlled [7], and parathyroidectomy is the only procedure known to halt the disease progression [4,8,9]. Furthermore, given the physical deformity and debility as the disease continues to evolve, SS patients are associated with a higher prevalence of psychiatric disorders than patients with ESRD alone [3]. 

In addition to the medical and genetic predisposition, the patient's socioeconomic status also plays an important role in the epidemiology and progression of the disease. Most reported cases are from regions with poor access to medical care [10], and it is rarely found in developed nations. This is likely because SS patients with adequate access to the healthcare system would have better control of their ESRD and hyperparathyroidism, thereby lowering their risk of developing the full SS phenotype [4]. 

Given the complexity of these patients, careful perioperative management and coordination are essential to ensure the safety of their care. In this report, we discuss a case of a female patient with ESRD and SS features admitted to the ICU for rapidly deteriorating acute respiratory failure. To our knowledge, this is the first documented case of SS in the United States. Given the patient’s poor respiratory status, difficult airway, and complex medical and psychosocial comorbidities, the perioperative anesthesia service (PAS) team, led by anesthesiologists with critical care background, was consulted for the airway and perioperative management. Furthermore, with increasing evidence indicating that a well-coordinated interdisciplinary perioperative service can improve long-term outcomes in surgical patients [11], the current case further highlights the benefit of having a dedicated PAS team in perioperative optimization, emergency planning, and coordination for medically complex patients as well.

## Case presentation

**Figure 1:**
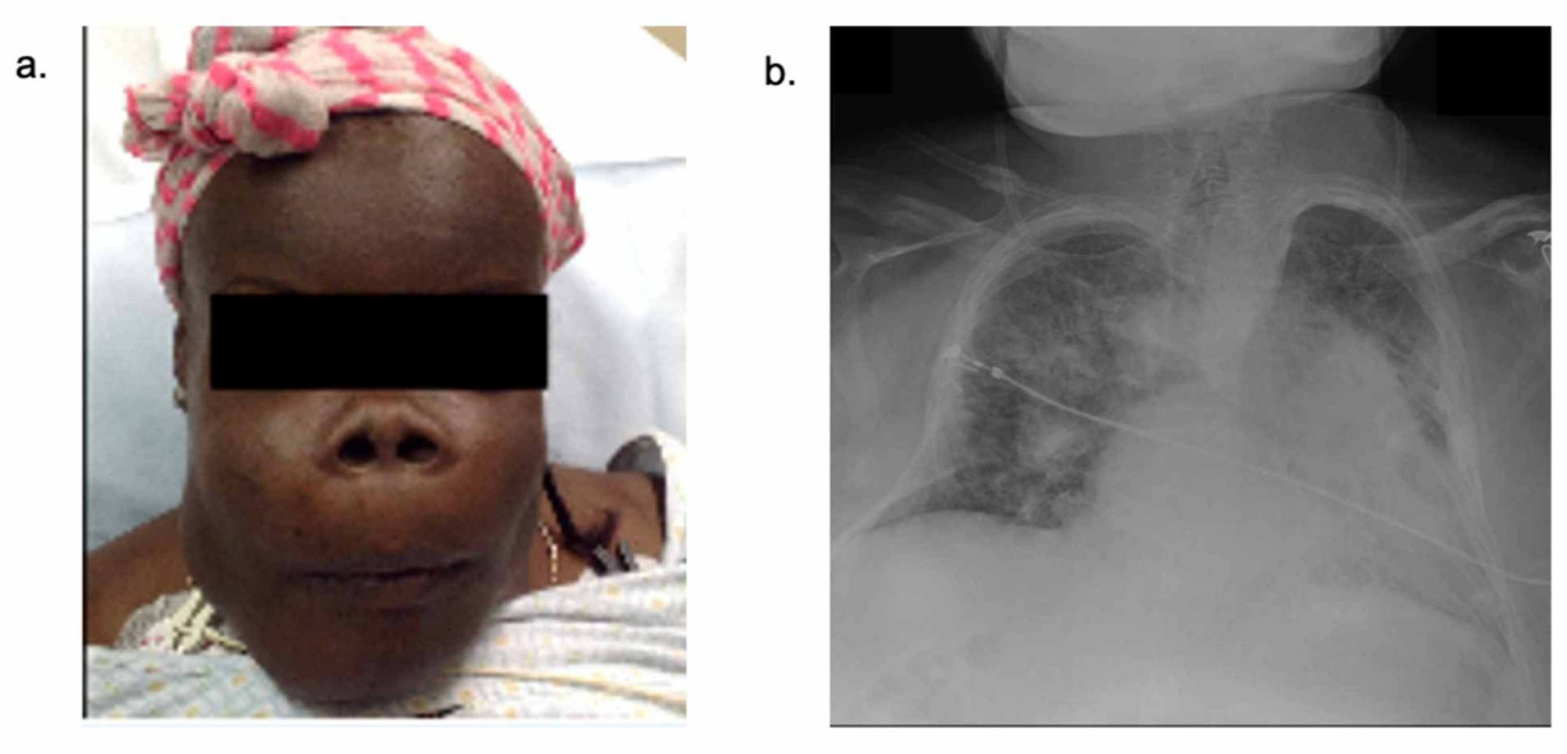
Patient initial presentation on admission Presentation on current admission: a) physical exam notable for severe midface hypertrophy, large forward-facing nostrils, and limited range of motion of the neck; b) chest X-ray on admission demonstrated bilateral restricted lung fields and severe pulmonary edema secondary to hypervolemia

The patient was a 37-year-old African American female with a history of ESRD and poorly controlled hyperparathyroidism. She also had a history of kidney transplant at 18 years of age, which had been complicated by host-versus-graft rejection at age 22 leading to hemodialysis dependency. She presented with acute exacerbation of respiratory failure due to chronic restricted lung disease, pulmonary edema, and hypervolemia in the setting of a malfunctioning dialysis fistula and missing three hemodialyses (Figure 1).

**Figure 2:**
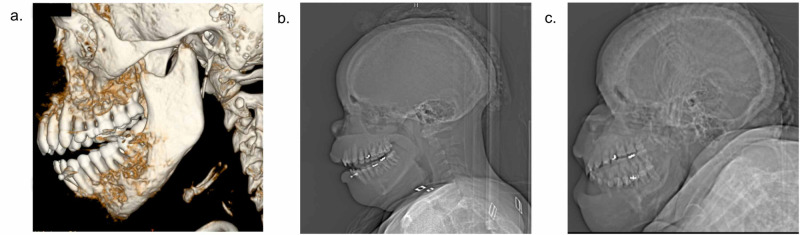
CT face and head demonstrate the patient's distorted anatomy and disease progression CT face (a) and head (b) with reconstruction when the patient was at the age of 28 demonstrated diffuse enlargement of bilateral mandibles, maxillas, hard palate, orbital walls, and bony thickening of the calvarium and skull base, with superimposed bony permeative change and periosteal reaction. The decreasing size of bilateral maxillary sinuses was also noted, secondary to bony overgrowth of the maxilla. (c) current patient facial features at age 37 with recent CT head showing diffuse osseous expansion, heterogeneous mineralization, and multifocal rounded foci of sclerosis and lucency, most prominent in the frontal calvarium and facial bone CT: computed tomography

The patient’s medical history also included progressive gum hypertrophy and outward prominence and deformity of her cheek and jaw (Figures 2a-2b) starting at age 28. Her PTH levels had been severely elevated at >2500 pg/ml (normal range: 10-80 pg/ml) at that time. Over the years, she had developed osteopenia, weakness, and bone pain in all four extremities, and dyspnea requiring home oxygen. Her physical activity had deteriorated as she developed neuropathy and had experienced frequent falls. Ultimately, she had become wheelchair-bound and unable to care for herself. 

Her dyspnea worsened on hospital day two despite aggressive hemodialysis and non-invasive respiratory support. After she rapidly desaturated on high flow oxygen, a code for acute respiratory distress was called. Her oxygenation was subsequently stabilized on nasal bilevel positive airway pressure (BiPAP) support, but she had to be transferred to the ICU for further monitoring. The PAS team was consulted for airway management and long-term planning.

Her airway exam was notable for a deformed mandible and maxilla, thick and severely flexed neck with poor surface anatomy, limited mouth opening, and a Mallampati score of IV. Recent imaging showed marked deformity of facial bones (Figure 2c). She also appeared fatigued, and her affect was flat. Given this exam, oral intubation was determined to be challenging. The otolaryngology (ENT) team was consulted by the PAS team for a flexible fiberoptic scope examination of the airway, which demonstrated an anteriorly displaced larynx accessible from the left nasopharynx passage. This route was deemed suitable for transnasal intubation. 

A comprehensive multidisciplinary airway management plan, involving the ICU, PAS, ENT, and cardiothoracic surgery teams, was created. First, given the patient had a poor respiratory reserve, the ICU team was in constant communication with all the involving services, and would have a low threshold for escalating to endotracheal intubation if the patient showed early signs of decompensation. In preparation for an awake transnasal fiberoptic intubation attempt by the PAS team, the cardiothoracic surgery team would first pre-cannulate the patient for potential extracorporeal membrane oxygenation (ECMO). In case of unsuccessful intubation, the patient could then be converted to ECMO to avoid life-threatening hypoxia. This bridge-oxygenation would allow the standby ENT team to perform a tracheostomy in a controlled environment. 

After communicating the plan with the patient, she elected to continue hemodialysis, agreed with intubation and ECMO but declined tracheotomy because it could impede her ability to speak. The patient also wished to remain full code, despite knowing that she would not have a full range of resuscitation options and staying on ECMO would not be a long-term solution. Palliative care and ethics teams were brought in by the PAS and the ICU team to further assist with the patient’s care given the inconsistency in her decision-making. After multiple multidisciplinary goals-of-care discussions amongst the patient, her family, and the care teams, it was agreed upon that if she deteriorated and failed to be intubated, comfort measures would be offered.

Fortunately, after five days of aggressive hemodialysis, the patient’s condition improved and she was weaned off BiPAP. She returned to her baseline respiratory status, and her malfunctioned fistula was exchanged by interventional radiology. Representative blood gas values throughout her hospital stay are shown in Table 1. On the day of discharge, the patient agreed to the follow-up plan but declined outpatient elective parathyroidectomy and tracheostomy.

**Table 1:** Representative venous blood gas during patient’s current admission Representative venous blood gas during patient’s current admission: on the day of admission, day of ICU transfer, and day of discharge ICU: intensive care unit; pCO_2_: CO_2_ partial pressure; pO_2_: O_2_ partial pressure; HCO_3_: bicarbonate concentration; BiPAP: bilevel positive airway pressure

Venous blood gas (reference range)	On the day of admission (with high flow O_2 _nasal cannula)	On the day of ICU transfer (with BiPAP)	On the day of discharge (with 2L O_2_ nasal cannula)
pH (7.32-7.42)	7.247 (L)	7.156 (L)	7.253 (L)
pCO_2_ (40-50 mmHg)	74.9 (H)	82.7 (H)	64.3 (H)
pO_2 _ (35-45 mmHg)	63.4 (H)	85.0 (H)	75.0 (H)
HCO_3_ (22-26 mEq/L)	31.5 (H)	29.5 (H)	27.4 (H)
Base excess (0-3 mEq/L)	4.6 (H)	1.4	1

## Discussion

SS is a rare but devastating condition in a subset of patients with ESRD. Likely a severe form of renal osteodystrophy [12], SS is characterized by a distinct constellation of anatomical and physiological features as seen in our patient [1-3,13]. Given her comorbidities, perioperative coordination facilitated by the PAS team was an essential part of her care plan. Although she had received medical care since childhood, her renal disease had been inadequately managed due to missed hemodialyses, which had directly contributed to her recurrent fluid overload and respiratory failure. She also had not undergone parathyroidectomy, the only procedure that is known to halt the progression of her disease [4,8,9], which had resulted in her difficult airway anatomy [13].

One of the responsibilities for the PAS team is to evaluate, coordinate, and assist with difficult airway management in a critical care or perioperative setting. When this patient first presented with severe acute respiratory distress, an important contribution by the PAS team was to derive a well-thought-out airway plan. With the information provided by the ENT team, the PAS team could potentially perform an awake transnasal fiberoptic intubation if this patient started to decompensate. Although her nasopharynx passage was seemingly straightforward, the PAS team recognized the potential factors that could complicate the intubation process, such as the patient becoming uncooperative, aspiration, or airway bleeding. If transnasal intubation was unsuccessful, this patient’s condition would pose a significant challenge for ventilation, because the conventional difficult airway algorithm was likely inadequate for her during an emergency [14]; the patient’s poor respiratory reserve and distorted anatomy precluded bag-mask ventilation or the use of a supraglottic airway, and her limited neck mobility and access also made oral endotracheal intubation or invasive airway challenging and unsafe. Taking this into consideration, ECMO could be a viable bridging option to a surgical airway, in an emergent situation of acute respiratory failure with a “cannot-intubate, cannot-ventilate” complex airway. Indeed, studies have demonstrated that EMCO can safely support patients with severe acute respiratory distress syndrome [15]. It has also been suggested that ECMO is an effective means of oxygenation in situations of airway obstruction [16]. 

Besides assisting with airway management and coordinating care for emergency situations, the PAS team is also valuable in assisting patients with their decision-making process. In this case, the patient’s psychosocial history was complex and played an important role in her care. It was unclear why this patient elected to forgo life-saving therapies, such as tracheostomy and parathyroidectomy. One explanation was the patient’s potential distrust with the medical establishment due to a failed kidney transplant and frequent admissions for malfunctioning dialysis fistula. Furthermore, with her long-term disability and lack of social support, the patient was at risk for undiagnosed psychiatric conditions, which could impact her decision-making ability. This is consistent with previous reports that SS patients have a high prevalence of depression and anxiety [3,10]. Recognizing these potential psychosocial factors, the PAS team worked closely with the primary team to communicate plans with the patient, promptly asked palliative care and ethics for assistance, and facilitated goals-of-care discussions for the patient. This ultimately yielded a final patient-driven care plan that was agreed upon by all concerned parties.

The current report has some limitations. It is confined to one patient with a rare condition, and some of the sources described, such as ECMO bridge-oxygenation, might not be readily available in some facilities. But this sequence of comprehensive evaluation and coordination has demonstrated that when serving patients with rare and complex medical conditions, having an early evaluation by a PAS team is essential for resource allocation and perioperative planning. Furthermore, the positive outcome of this case further supports that, besides physiological considerations, a psychological evaluation is a valuable part of perioperative management for patients with a long-term disability to ensure optimal and appropriate care.

## Conclusions

In summary, the current report highlights an innovative airway plan for a complex, potentially “cannot-intubate, cannot-ventilate” airway, and the role of the PAS team in facilitating perioperative care for a patient with SS, a rare debilitating disease with complex comorbidities, in a critical care and perioperative setting. This report further supports the notion that implementing a comprehensive perioperative care plan for medically complex patients can improve the safety of their care, and positively impact their recovery and outcomes.
